# Post-COVID-19 Condition in Hospitalized Survivors After 1 Year of Infection During the Alpha- and Delta-variant Dominant Waves in Japan: COVID-19 Recovery Study II

**DOI:** 10.2188/jea.JE20240179

**Published:** 2025-07-05

**Authors:** Yoko Muto, Mariko Hosozawa, Miyuki Hori, Arisa Iba, Shuhei Maruyama, Shinichiro Morioka, Katsuji Teruya, Takeshi Nishida, Toshiyuki Harada, Hideki Yoshida, Satoshi Miike, Akira Kawauchi, Hideaki Kato, Junji Hatakeyama, Shigeki Fujitani, Tomohiro Asahi, Kensuke Nakamura, Yuichi Sato, Taku Oshima, Futoshi Nagashima, Kohei Ota, Tatsuya Fuchigami, Nobuyuki Nosaka, Hiroshi Kamijo, Takeshi Hattori, Hayato Taniguchi, Hiroyasu Iso

**Affiliations:** 1 Institute for Global Health Policy Research (iGHP), Bureau of International Health Cooperation, National Center for Global Health and Medicine, Tokyo, Japan; 2 Department of Emergency and Critical Care Medicine, Kansai Medical University Medical Center, Osaka, Japan; 3 Disease Control and Prevention Center, National Center for Global Health and Medicine, Tokyo, Japan; 4 AIDS Clinical Center, National Center for Global Health and Medicine, Tokyo, Japan; 5 Division of Trauma and Surgical Critical Care, Osaka General Medical Center, Osaka, Japan; 6 Department of Acute Medicine and Intensive Care Medicine, Osaka University Hospital, Osaka, Japan; 7 Center for Respiratory Disease, Department of Respiratory Medicine, Japan Community Healthcare Organization Hokkaido Hospital, Hokkaido, Japan; 8 Department of Emergency and Critical Care Medicine, St. Marianna University Yokohama Seibu Hospital, Kanagawa, Japan; 9 Department of Critical Care and Emergency Medicine, Japanese Red Cross Maebashi Hospital, Gunma, Japan; 10 Infection Prevention and Control Department, Yokohama City University Hospital, Kanagawa, Japan; 11 Department of Emergency and Critical Care Medicine, Osaka Medical and Pharmaceutical University, Osaka, Japan; 12 Department of Emergency and Critical Care Medicine, St. Marianna University School of Medicine, Kanagawa, Japan; 13 Department of Cardiology, Naha City Hospital, Okinawa, Japan; 14 Department of Emergency and Critical Care Medicine, Hitachi General Hospital, Ibaraki, Japan; 15 Department of Critical Care Medicine, Yokohama City University Hospital, Kanagawa, Japan; 16 Department of Emergency and Critical Care, Tokyo Metropolitan Tama Medical Center, Tokyo, Japan; 17 Department of Emergency and Critical Care Medicine, Chiba University Graduate School of Medicine, Chiba, Japan; 18 Tajima Emergency and Critical Care Medical Center, Toyooka Public Hospital, Hyogo, Japan; 19 Department of Emergency and Critical Care Medicine, Graduate School of Biomedical and Health Sciences, Hiroshima University, Hiroshima, Japan; 20 Department of Anesthesiology and Intensive Care Medicine, University of the Ryukyus Hospital, Okinawa, Japan; 21 Department of Intensive Care Medicine, Tokyo Medical and Dental University, Tokyo, Japan; 22 Department of Emergency and Critical Care Medicine, Shinshu University School of Medicine, Nagano, Japan; 23 Department of Respiratory Medicine, National Hospital Organization, Hokkaido Medical Center, Hokkaido, Japan; 24 Advanced Critical Care and Emergency Center, Yokohama City University Medical Center, Yokohama, Japan

**Keywords:** COVID-19, post-COVID-19 condition, mental health, the Alpha variant, the Delta variant

## Abstract

**Background:**

Evidence of post-COVID-19 condition (PCC) in the Alpha- and Delta-variant dominant waves is limited.

**Methods:**

In a nationwide multicenter cohort study in collaboration with 20 hospitals, we collected data using self-administered questionnaires and electronic medical records of participants aged 20 or more diagnosed with COVID-19, hospitalized between April 1, 2021 and September 30, 2021, and discharged alive. Descriptive statistics were analyzed for PCC and mental health (HADS anxiety and depression scores), comparing Alpha- and Delta-variant dominant waves.

**Results:**

We analyzed 1,040 patients (median age, 57 [IQR 49–66] years; men, 66.2%). Of the respondents, 45.4% had at least one PCC symptom 1 year after infection. The common symptoms included dyspnea (20.7%), fatigue/malaise (17.6%), muscle weakness (15.4%), decrease in concentration (13.4%), and sleep disorder (13.3%), followed by brain fog (8.4%). Among patients with PCC, 14.0% had anxiety (HADS-Anxiety ≥11), and 18.6% had depression (HADS-Depression ≥11), with four times higher proportions than those without PCC; only small variations by age, sex, and waves were observed. Associated factors for PCC were age 40 years or over, women, severity of COVID-19 during hospitalization, ex-smokers who quit smoking before COVID-19 infection and being infected during the Delta-variant dominant wave.

**Conclusion:**

The study described the prevalence of PCC, associated factors, and mental health of COVID-19 survivors hospitalized during the Alpha- and Delta-variant dominant waves in Japan. Further follow-up will be conducted to examine the longer-term impact of COVID-19 on PCC, complications, daily life, and socioeconomic status.

## INTRODUCTION

The pandemic of the coronavirus disease 2019 (COVID-19) has notably affected human health and daily life. Even after infection, COVID-19 induces persistent symptoms, such as fatigue, malaise, and dyspnea, as well as the appearance of symptoms not present in the acute phase, such as brain fog and hair loss. They are reported globally as ‘post-COVID-19 condition (PCC)’, ‘post-acute sequelae of SARS-CoV-2 infection (PASC)’, or ‘Long COVID’. More than 65 million individuals worldwide are believed to have PCC, and thus, clarifying PCC and taking appropriate action are urgent global issues.^1^

Moreover, PCC includes multifaceted symptoms associated with the heart, lungs, gastrointestinal tract, pancreas, immune system, neurological system, blood vessels, kidneys, spleen, liver, and reproductive system.^1^^,^^2^ In some patients, these symptoms last for years,^3^^–^^6^ which could have long-lasting adverse effects on daily life.^7^ Most of the studies examining the situations of PCC after 1 or more years from the infection were based on the infection in 2020.^3^^–^^6^ The number of studies is very limited to examine PCC from the infection in 2021 and later.^8^^–^^11^ The Alpha-variant was prevalent in spring 2021, and the Delta-variant was in summer 2021 in Japan. The COVID-19 vaccination started for medical workers in February 2021, older people in April 2021, and those aged over 18 years in June 2021. Fewer people, especially younger ones, were vaccinated during the Alpha- and Delta-variant dominant waves. During the Delta-variant dominant wave, the maximum number of seriously ill COVID-19 patients receiving treatment per day exceeded 2,000 in September 2021.^12^ Among the studies on the infection in 2021 and later,^8^^–^^11^ two of them compared PCC between Alpha- and Delta-variant dominant periods among people living in Belgium^8^ and Hiroshima, Japan,^10^ in which only 1% and 37% of the patients, respectively, had been hospitalized. Investigating the long-term prognosis of hospitalized patients infected during the Alpha- and Delta-variant dominant periods is needed to offer better health care support.

We launched a longitudinal multicenter survey, the COVID-19 Recovery Study II (CORES II), between August 2022 and September 2022. This study described the study design, prevalence of PCC, associated factors for PCC, and mental health 1 year after COVID-19 during the Alpha- and Delta-variant dominant waves.

## METHODS

### Study design and participants

**Figure 1.**
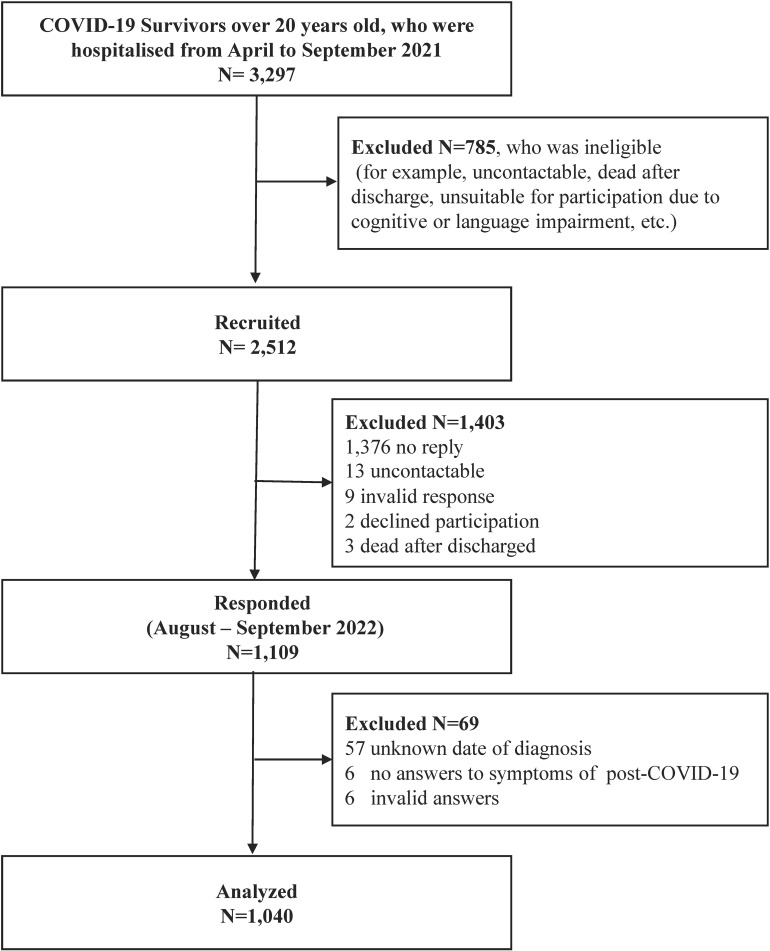
Selection flow of the study population. This flow chart shows the recruitment and selection of participants. The median number of days from the onset of COVID-19 to the answer date was 422 (range, 316–565) days. COVID-19, coronavirus disease 2019.

The CORES II study is a nationwide multicenter cohort study in collaboration with 20 regional base medical institutions from 11 prefectures that provided inpatient care for patients with COVID-19 (eMaterial 1). Each institution participated in 20 medical department units: 15 departments of emergency medicine or intensive care unit (ICU) and five departments of respiratory medicine or others. The inclusion criteria for CORES II were patients aged 20 years or older diagnosed with COVID-19, hospitalized between April 1, 2021 and September 30, 2021, and discharged alive from collaborating institutions. Among 3,297 patients who met these criteria, those who could not be contacted (eg, died after discharge from the hospital or current address unknown), those who were deemed ineligible due to language or cognitive difficulties, and those who were turned down when contacted in advance by telephone were excluded (Figure 1). An invitation letter for this study was sent to 2,512 eligible patients, and those who consented to participate completed a paper-based questionnaire between August 1, 2022, and September 30, 2022.

### Questionnaire

The after-1-year questionnaire survey included questions regarding the symptoms after being infected by COVID-19. The presence or absence and the duration of the following 26 symptoms, selected from the International Severe Acute Respiratory and Emerging Infection Consortium (ISARIC) follow-up questionnaire,^13^ were asked: fever, fatigue/malaise, sore throat, rhinorrhea, cough, dyspnea, chest pain, palpitation, dysgeusia, anosmia, headache, joint pain/joint swelling, myalgia, muscle weakness, anorexia, nausea/vomiting, abdominal pain, sleep disorder, decrease in concentration, brain fog, hair loss, skin rash, eye symptoms, dizziness, erectile dysfunction (for men), and menstrual changes (for women).

Mental health status was measured using the Japanese version of the Hospital Anxiety and Depression Scale (HADS).^14^ Demographic information, such as age and sex, body mass index (BMI) at admission, newly diagnosed disease after COVID-19 infection, hospital admission after discharge due to COVID-19-related or other conditions, re-infection with COVID-19, and COVID-19 vaccination status, were also collected. Other questions included socioeconomic status (years of education, household income, occupation, and employment status), cohabitation and marital status, health behaviors (eg, smoking, drinking, physical activity, sedentary habits, sleep duration), and change in work situation since COVID-19 infection.

### Clinical data

The clinical data of the participants were obtained from their medical records. The collected data included the date of COVID-19 diagnosis, dates of admission and discharge, and comorbidities. Physical condition on admission (symptoms, height, weight, body temperature, blood pressure, pulse rate, respiratory rate, oxygen saturation, measurement conditions, and consciousness level), hematological, and chest X-ray findings were collected. We also collected data on clinical course and treatment, ICU admission, complications, use of renal replacement therapy or dialysis, plasma apheresis, and medications (COVID-19 medications, steroids, and anticoagulants), information regarding the place of discharge (home, transferred to rehabilitation hospital, transferred to advanced medical care hospital, or transferred to nursing homes), and oxygen administration upon discharge.

### Collection of data and management

Our study data were collected and managed using Research Electronic Data Capture (REDCap),^15^ a secure web-based data capture application hosted at the JCRAC Data Center of the National Center for Global Health and Medicine.

### Statistical analysis

Descriptive statistics were performed on the respondents’ characteristics, PCC symptoms, and HADS and reported as median with interquartile range (IQR) or range for continuous variables and the numbers with percentages for categorical variables. We defined PCC as symptoms that appeared within 3 months of diagnosis and persisted at the time of response to the after-1-year survey referring to the World Health Organization’s (WHO) definition of post-COVID-19 condition (PCC).^16^ We used the WHO clinical progression scale to determine the severity at the time of hospitalization for COVID-19: moderate as required no oxygen therapy or required oxygen by mask or nasal prongs; severe as required oxygen by non-invasive ventilation or high flow, or required mechanical ventilation or extracorporeal membrane oxygenation (ECMO).^17^ The follow-up periods were estimated from the COVID-19 diagnosis to the date of answering the questionnaire. Regarding the HADS subscale (anxiety/depression), we applied a cut-off score of 0–7 in the normal range, 8–10 as suggestive anxiety/depressive symptoms, and 11 or higher as anxiety/depressive symptoms.

IBM SPSS Statistics version 28 (IBM Corp., Armonk, NY, USA) was used for all analyses. The characteristics and proportion of PCC symptoms of the Alpha-variant and Delta-variant dominant waves were compared using Pearson’s χ^2^ tests or Fisher’s exact tests for categorical variables and Mann-Whitney-U-tests for continuous variables. Multivariable logistic regression analysis was used to investigate associated factors for PCC, HADS-Anxiety, and HADS-Depression. *P*-value <0.05 (two-tailed) was considered statistically significant.

### Ethics declarations

All procedures involving human participants were reviewed and approved by the institutional review boards of the National Center for Global Health and Medicine (approval number: NCGM-S-004471) and the ethics committees of all participating institutions. Written informed consent was obtained from all the participants.

## RESULTS

A total of 1,109 participants responded, with a response rate of 44.1% (1,109/2,512). The questionnaire’s response rate did not vary materially among the collaborating centers (eTable 1). We included 1,040 participants in the analysis, leaving out 69 patients who did not respond about symptoms of PCC, whose COVID-19 diagnosis date was unknown, or whose answers were invalid (Figure 1).

Table 1 presents the characteristics of the study participants by wave. Overall, the median age of the total participants was 57, and 46.3% were 40–59 years old; 66.2% were men; 63.9% had preexisting comorbidities, such as hypertension (33.6%), diabetes mellitus (22.7%), and dyslipidemia (18.3%); and 47.1% had severe COVID-19 during hospitalization. Compared to patients with the Alpha-variant dominant wave, those with the Delta-variant dominant wave were younger, more likely to be women, more educated, regular employees, had fewer comorbidities, had moderate severity of COVID-19 during hospitalization, and were less unemployed.

**Table 1.** Characteristics of the study participants by waves

	Alpha-variant dominant wave^a^ (*n* = 490)		Delta-variant dominant wave^b^ (*n* = 550)		*P*-value^*^

	Median, *n*	[Range, IQR], (%)	Median, *n*	[Range, IQR], (%)	
Follow up duration, median [range] days^c^	489	[404–565]	383	[316–451]	<0.001
Age, median [IQR] years	61	[53–72]	54	[46–61]	<0.001
Age group, years					
20–39	32	(6.5)	75	(13.6)	<0.001
40–59	183	(37.3)	298	(54.2)	
60–79	231	(47.1)	152	(27.6)	
≥80	35	(7.1)	18	(3.3)	
Missing	9	(1.8)	7	(1.3)	
Sex					
Men	344	(70.2)	344	(62.5)	0.01
Women	146	(29.8)	206	(37.5)	
Comorbidities before infection					
No	147	(30.0)	228	(41.5)	<0.001
Yes	343	(70.0)	322	(58.5)	
Hypertension	204	(41.6)	145	(26.4)	
Diabetes mellitus	132	(26.9)	104	(18.9)	
Dyslipidemia	113	(23.1)	77	(14.0)	
Respiratory diseases^d^	50	(10.2)	55	(10.0)	
Malignant tumor	27	(5.5)	32	(5.8)	
Mental disorders^e^	16	(3.3)	28	(5.1)	
Cardiovascular diseases^f^	18	(3.7)	24	(4.4)	
Severity of COVID-19 during hospitalization					
Moderate	224	(45.7)	326	(59.3)	<0.001
Severe	266	(54.3)	224	(40.7)	
BMI at admission, kg/m^2^					
<18.5	12	(2.4)	16	(2.9)	0.49
18.5–24.9	218	(44.5)	229	(41.6)	
≥25.0	250	(51.0)	303	(55.1)	
Missing	10	(2.0)	2	(0.4)	
Smoking status					
Non-smoker	203	(41.4)	239	(43.5)	0.33
Ex-smoker who quit before COVID-19 infection	210	(42.9)	213	(38.7)	
Ex-smoker who quit after COVID-19 infection	34	(6.9)	37	(6.7)	
Current smoker	36	(7.3)	55	(10.0)	
Missing	7	(1.4)	6	(1.1)	
Drinking status					
Never	98	(20.0)	134	(24.4)	0.31
Rarely	110	(22.4)	120	(21.8)	
1–3 times a month	48	(9.8)	60	(10.9)	
1–2 times a week	55	(11.2)	58	(10.5)	
3–4 times a week	44	(9.0)	56	(10.2)	
5–6 times a week	45	(9.2)	49	(8.9)	
Every day	82	(16.7)	66	(12.0)	
Missing	8	(1.6)	7	(1.3)	
Physical activity per week					
Rarely	268	(54.7)	333	(60.5)	0.15
1–2 hours	95	(19.4)	112	(20.4)	
3–4 hours	58	(11.8)	56	(10.2)	
5–6 hours	40	(8.2)	27	(4.9)	
≥7 hours	22	(4.5)	21	(3.8)	
Missing	7	(1.4)	1	(0.2)	
Sedentary hours per day					
<1 hour	11	(2.2)	16	(2.9)	0.36
1–2 hours	41	(8.4)	65	(11.8)	
3–4 hours	122	(24.9)	152	(27.6)	
5–6 hours	134	(27.3)	132	(24.0)	
7–8 hours	97	(19.8)	106	(19.3)	
9–10 hours	52	(10.6)	53	(9.6)	
≥11 hours	27	(5.5)	23	(4.2)	
Missing	6	(1.2)	3	(0.5)	
Sleep hours per day					
<5 hours	42	(8.6)	54	(9.8)	0.25
5 hours	82	(16.7)	95	(17.3)	
6 hours	166	(33.9)	215	(39.1)	
7 hours	117	(23.9)	119	(21.6)	
8 hours	58	(11.8)	48	(8.7)	
≥9 hours	15	(3.1)	11	(2.0)	
Missing	10	(2.0)	8	(1.5)	
Occupation before infection					
Managerial occupations	91	(18.6)	87	(15.8)	0.004
Professional and technical occupations	76	(15.5)	90	(16.4)	
Administration	62	(12.7)	69	(12.5)	
Service industry	46	(9.4)	67	(12.2)	
Sales	19	(3.9)	24	(4.4)	
Security occupation	6	(1.2)	8	(1.5)	
Production process	5	(1.0)	19	(3.5)	
Agriculture, forestry and fisheries	1	(0.2)	3	(0.5)	
Transportation and machine operation	17	(3.5)	11	(2.0)	
Construction and mining	16	(3.3)	31	(5.6)	
Cleaning, packaging	8	(1.6)	14	(2.5)	
Students and homemaker	42	(8.6)	50	(9.1)	
Other	20	(4.1)	30	(5.5)	
Unemployed	77	(15.7)	44	(8.0)	
Missing	4	(0.8)	3	(0.5)	
Employment before infection					
Regular employee	207	(42.2)	265	(48.2)	0.003
Self-employed	69	(14.1)	75	(13.6)	
Part-time	87	(17.8)	112	(20.4)	
Students and housewives	38	(7.8)	45	(8.2)	
Unemployed	78	(15.9)	42	(7.6)	
Others	4	(0.8)	7	(1.3)	
Missing	7	(1.4)	4	(0.7)	
Cohabit					
No	100	(20.4)	111	(20.2)	0.91
Yes	385	(78.6)	435	(79.1)	
Missing	5	(1.0)	4	(0.7)	
Married					
No	164	(33.5)	199	(36.2)	0.47
Yes	312	(63.7)	344	(62.5)	
Missing	14	(2.9)	7	(1.3)	
Years of education					
≤12	253	(51.6)	249	(45.3)	0.03
≥13	228	(46.5)	296	(53.8)	
Do not want to answer	2	(0.4)	3	(0.5)	
Missing	7	(1.4)	2	(0.4)	
Household income in 2021, yen					
<4 million	212	(43.3)	205	(37.3)	0.06
≥4 million	238	(48.6)	284	(51.6)	
Prefer not to answer	34	(6.9)	55	(10.0)	
Missing	6	(1.2)	6	(1.1)	
Changed work since infection					
No	402	(82.0)	458	(83.3)	0.98
Yes	75	(15.3)	85	(15.5)	
Missing	13	(2.7)	7	(1.3)	

BMI, body mass index; COVID-19, coronavirus disease 2019; IQR, interquartile range.

These data represent the numbers and percentages of participants, and the median and IQR or range. The severity of COVID-19 during hospitalization was classified into two categories: moderate (no oxygen therapy or oxygen by mask or nasal prongs) and severe (oxygen by non-invasive ventilation or high flow, mechanical ventilation, or extracorporeal membrane oxygenation).

^a^Between 24 February 2021 and 30 June 2021.

^b^Between 1 July 2021 and 28 September 2021.

^c^Defined as duration from the onset of COVID-19 to answer date.

^d^Interstitial lung disease, asthma, and chronic obstructive pulmonary disease.

^e^Depression, anxiety, and schizophrenia.

^f^Myocardial infarction and heart failure.

^*^P-values for group differences were obtained using Pearson’s χ^2^ tests excluding missing data for categorical variables and Mann-Whitney U test for continuous variables.

**Figure 2.**
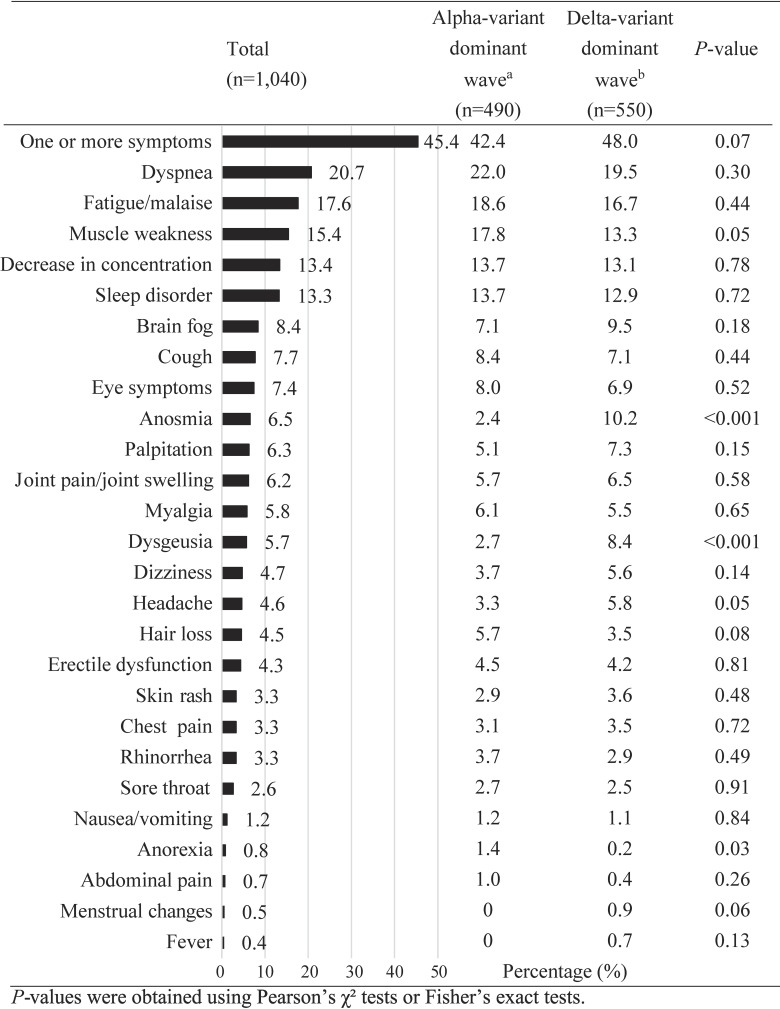
Proportions of post-COVID-19 symptoms 1 year after COVID-19 infection This figure shows the percentage of PCC symptoms according to the overall, the Alpha-variant dominant, and the Delta-variant dominant waves. ^a^Between February 24, 2021 and June 30, 2021. ^b^Between July 1, 2021, and September 28, 2021. COVID-19, coronavirus disease 2019; PCC, post-COVID-19 condition.

Figure 2 illustrates the proportion of PCC 1 year after COVID-19 infection: 472 (45.4%) reported having one or more symptoms. In total, the common symptoms included dyspnea (*n* = 215, 20.7%), fatigue/malaise (*n* = 183, 17.6%), muscle weakness (*n* = 160, 15.4%), a decrease in concentration (*n* = 139, 13.4%), and sleep disorders (*n* = 138, 13.3%) followed by brain fog (*n* = 87, 8.4%). The proportion of PCC did not differ largely between the Alpha- and Delta-variant waves (42.4% vs 48.0%, respectively). Higher proportions of anosmia and dysgeusia were observed in the Delta-variant dominant wave, while the opposite trend was seen for anorexia in the Alpha-variant dominant waves.

Characteristics of patients were compared between patients with and without PCC (Table 2). Compared with patients without PCC, those with PCC were more likely to be aged 40 years and older, have comorbidities before infection, have severe cases of COVID-19 during hospitalization, be more ex-smokers who quit before COVID-19 infection, physically inactive, more sedentary, sleep less than 5 hours, and had changed work since COVID-19 infection.

**Table 2.** Characteristics of study participants according to the presence or absence of post-COVID-19 condition

	PCC (+) (*n* = 472)		PCC (−) (*n* = 568)		*P*-value^*^

	Median, *n*	[Range, IQR], (%)	Median, *n*	[Range, IQR], (%)	
Follow up duration, median [range] days	418	[316–565]	427	[327–545]	0.78
Wave					
Alpha-variant dominant	208	(49.6)	282	(44.1)	0.07
Delta-variant dominant	264	(50.4)	286	(55.9)	
Age, median [IQR] years	59.0	[51–67]	57.0	[47–67]	0.04
Age group, years					
20–39	25	(5.3)	82	(14.4)	<0.001
40–59	234	(49.6)	247	(43.5)	
60–79	182	(38.6)	201	(35.4)	
≥80	23	(4.9)	30	(5.3)	
Missing	8	(1.7)	8	(1.4)	
Sex					
Men	307	(65.0)	381	(67.1)	0.49
Women	165	(35.0)	187	(32.9)	
Comorbidities before infection					
No	151	(32.0)	224	(39.4)	0.01
Yes	321	(68.0)	344	(60.6)	
Severity of COVID-19 during hospitalization					
Moderate	210	(44.5)	340	(59.9)	<0.001
Severe	262	(55.5)	228	(40.1)	
BMI at admission, kg/m^2^					
<18.5	10	(2.1)	18	(3.2)	0.39
18.5–24.9	198	(41.9)	249	(43.8)	
≥25.0	260	(55.1)	293	(51.6)	
Missing	4	(0.8)	8	(1.4)	
Smoking status					
Non-smoker	184	(39.0)	258	(45.4)	0.003
Ex-smoker who quit before COVID-19 infection	214	(45.3)	209	(36.8)	
Ex-smoker who quit after COVID-19 infection	38	(8.1)	33	(5.8)	
Current smoker	31	(6.6)	60	(10.6)	
Missing	5	(1.1)	8	(1.4)	
Drinking status					
Never	116	(24.6)	116	(20.4)	0.61
Rarely	107	(22.7)	123	(21.7)	
1–3 times a month	50	(10.6)	58	(10.2)	
1–2 times a week	48	(10.2)	65	(11.4)	
3–4 times a week	45	(9.5)	55	(9.7)	
5–6 times a week	41	(8.7)	53	(9.3)	
Every day	59	(12.5)	89	(15.7)	
Missing	6	(1.3)	9	(1.6)	
Physical activity per week					
Rarely	295	(62.5)	306	(53.9)	0.04
1–2 hours	87	(18.4)	120	(21.1)	
3–4 hours	39	(8.3)	75	(13.2)	
5–6 hours	29	(6.1)	38	(6.7)	
≥7 hours	18	(3.8)	25	(4.4)	
Missing	4	(0.8)	4	(0.7)	
Sedentary hours per day					
<1 hour	8	(1.7)	19	(3.3)	0.01
1–2 hours	51	(10.8)	55	(9.7)	
3–4 hours	108	(22.9)	166	(29.2)	
5–6 hours	118	(25.0)	148	(26.1)	
7–8 hours	100	(21.2)	103	(18.1)	
9–10 hours	49	(10.4)	56	(9.9)	
≥11 hours	33	(7.0)	17	(3.0)	
Missing	5	(1.1)	4	(0.7)	
Sleep hours per day					
<5 hours	63	(13.3)	33	(5.8)	<0.001
5 hours	84	(17.8)	93	(16.4)	
6 hours	159	(33.7)	222	(39.1)	
7 hours	100	(21.2)	136	(23.9)	
8 hours	42	(8.9)	64	(11.3)	
≥9 hours	16	(3.4)	10	(1.8)	
Missing	8	(1.7)	10	(1.8)	
Occupation before infection					
Managerial occupations	91	(19.3)	87	(15.3)	0.21
Professional and technical occupations	71	(15.0)	95	(16.7)	
Administration	50	(10.6)	81	(14.3)	
Service industry	50	(10.6)	63	(11.1)	
Sales	22	(4.7)	21	(3.7)	
Security occupation	4	(0.8)	10	(1.8)	
Production process	11	(2.3)	13	(2.3)	
Agriculture, forestry and fisheries	0	(0.0)	4	(0.7)	
Transportation and machine operation	12	(2.5)	16	(2.8)	
Construction and mining	24	(5.1)	23	(4.0)	
Cleaning, packaging	8	(1.7)	14	(2.5)	
Students and homemaker	47	(10.0)	45	(7.9)	
Other	27	(5.7)	23	(4.0)	
Unemployed	54	(11.4)	67	(11.8)	
Missing	1	(0.2)	6	(1.1)	
Employment before infection					
Regular employee	209	(44.3)	263	(46.3)	0.74
Self-employed	69	(14.6)	75	(13.2)	
Part-time	84	(17.8)	115	(20.2)	
Students and housewives	43	(9.1)	40	(7.0)	
Unemployed	55	(11.7)	65	(11.4)	
Others	6	(1.3)	5	(0.9)	
Missing	6	(1.3)	5	(0.9)	
Cohabit					
No	93	(19.7)	118	(20.8)	0.64
Yes	376	(79.7)	444	(78.2)	
Missing	3	(0.6)	6	(1.1)	
Married					
No	164	(34.7)	199	(35.0)	0.90
Yes	299	(63.3)	357	(62.9)	
Missing	9	(1.9)	12	(2.1)	
Years of education					
≤12	240	(50.8)	262	(46.1)	0.13
≥13	226	(47.9)	298	(52.5)	
Do not want to answer or missing	6	(1.3)	8	(1.4)	
Household income in 2021, yen					
<4 million	193	(40.9)	224	(39.4)	0.43
≥4 million	228	(48.3)	294	(51.8)	
Prefer not to answer	45	(9.5)	44	(7.7)	
Missing	6	(1.3)	6	(1.1)	
Changed work since infection					
No	375	(79.4)	485	(85.4)	0.01
Yes	87	(18.4)	73	(12.9)	
Missing	10	(2.1)	10	(1.8)	

BMI, body mass index; COVID-19, coronavirus disease 2019; IQR, interquartile range; PCC, post-COVID-19 condition.

^*^P-values for group differences were obtained using Pearson’s χ^2^ tests excluding missing data for categorical variables and Mann-Whitney U test for continuous variables.

Table 3 shows multivariable odd ratios (ORs) and 95% confidence intervals (CIs) of PCC according to the selected variables. When mutually adjusted, the factors independently associated with PCC were higher ages compared to ages of 20–39, being a woman, having severe conditions of COVID-19 during hospitalization, ex-smoking before COVID-19 infection, being infected during Dela-variant dominant wave, and longer follow-up duration.

**Table 3.** Multivariable odd ratios and 95% confidence intervals of post-COVID-19 condition according to age, sex, and other characteristics

	Multivariable OR^a^	95% CI		*P*-value
Follow-up duration				
Days	1.01	1.00	1.01	0.03
Wave				
Alpha-variant dominant	1.00			
Delta-variant dominant	2.63	1.46	4.76	0.001
Age group, years				
20–39	1.00			
40–59	2.79	1.68	4.63	<0.001
60–79	2.57	1.51	4.37	<0.001
≥80	2.33	1.05	5.16	0.04
Sex				
Men	1.00			
Women	1.48	1.08	2.03	0.01
Comorbidities				
No	1.00			
Yes	1.08	0.75	1.56	0.68
Severity of COVID-19 during hospitalization				
Moderate	1.00			
Severe	1.85	1.36	2.53	<0.001
BMI at admission, kg/m^2^				
<18.5	1.00			
18.5–24.9	1.69	0.71	4.04	0.24
≥25.0	1.70	0.71	4.08	0.23
Smoking status				
Never-smoker	1.00			
Ex-smoker who quit before COVID-19 infection	1.41	1.04	1.92	0.03
Ex-smoker who quit after COVID-19 infection	1.54	0.89	2.66	0.12
Current smoker	0.81	0.48	1.34	0.41
Years of education				
≤12	1.00			
≥13	0.88	0.67	1.16	0.36
Household income, yen				
<4 million	1.00			
≥4 million	0.89	0.66	1.20	0.43
Prefer not to answer	1.42	0.86	2.35	0.17

CI, confidence interval; COVID-19, coronavirus disease 2019; BMI, body mass index; OR, odds ratio.

^a^Mutually adjusted for all variables listed.

Mental health status (HADS-Anxiety and Depression scores) 1 year after COVID-19 infection according to the presence or absence of PCC overall, by sex, and by age groups are presented in Table 4. The median HADS-Anxiety score was 5 (IQR, 3–9) among patients with PCC and 3 (IQR, 1–5) among those without it, and the proportion of HADS-Anxiety scores of 8 or more was 30.3% and 11.1%, respectively (19.8% total). The median HADS-Depression score was 6 (IQR, 3–10) among patients with PCC and 2 (IQR, 1–5) among those without it, and the proportion of HADS-Depression scores 8 or higher was 37.2% and 11.9%, respectively (23.5% total). The differences in mental status between the presence and absence of PCC did not vary materially by age and sex.

**Table 4.** Distribution of Hospital Anxiety and Depression Scale according to the presence or absence of post-COVID-19 condition overall, by age and sex

Overall	HADS-Anxiety					HADS-Depression				

	PCC (+)		PCC (−)		*P*-value	PCC (+)		PCC (−)		*P*-value
	(*n* = 472)		(*n* = 568)			(*n* = 472)		(*n* = 568)		
	
	Median/*n*	[IQR]/(%)	Median/*n*	[IQR]/(%)		Median/*n*	[IQR]/(%)	Median/*n*	[IQR]/(%)	
Score, median [IQR]	5	[3–9]	3	[1–5]		6	[3–10]	2	[1–5]	
Total score										
0–7	312	(66.1)	483	(85.0)	<0.001	283	(60.0)	482	(84.9)	<0.001
8–10	77	(16.3)	51	(9.0)		88	(18.6)	49	(8.6)	
≥11	66	(14.0)	12	(2.1)		88	(18.6)	19	(3.3)	
Missing	17	(3.6)	22	(3.9)		13	(2.8)	18	(3.2)	

Stratified by age group										
20–39 years old										
0–7	13	(52.0)	70	(85.4)	<0.001	14	(56.0)	71	(86.6)	<0.001
8–10	3	(12.0)	9	(11.0)		5	(20.0)	9	(11.0)	
11 or more	9	(36.0)	3	(3.7)		6	(24.0)	2	(2.4)	
Missing	0	(0.0)	0	(0.0)		0	(0)	0	(0)	
40–59 years old										
0–7	157	(67.1)	213	(86.2)	<0.001	141	(60.3)	219	(88.7)	<0.001
8–10	37	(15.8)	30	(12.1)		39	(16.7)	19	(7.7)	
11 or more	35	(15.0)	1	(0.4)		49	(20.9)	6	(2.4)	
Missing	5	(2.1)	3	(1.2)		5	(2.1)	3	(1.2)	
60–79 years old										
0–7	120	(65.9)	170	(84.6)	<0.001	108	(59.3)	164	(81.6)	<0.001
8–10	33	(18.1)	10	(5.0)		36	(19.8)	18	(9.0)	
11 or more	20	(11.0)	7	(3.5)		31	(17.0)	11	(5.5)	
Missing	9	(4.9)	14	(7.0)		7	(3.8)	8	(4.0)	
80 years and over										
0–7	15	(65.2)	26	(86.7)	0.22	16	(69.6)	24	(80.0)	0.20
8–10	3	(13.0)	1	(3.3)		4	(17.4)	3	(10.0)	
11 or more	2	(8.7)	1	(3.3)		2	(8.7)	0	(0)	
Missing	3	(13.0)	2	(6.7)		1	(4.3)	3	(10.0)	

Stratified by sex										
Men										
0–7	212	(69.1)	327	(85.8)	<0.001	195	(63.5)	326	(85.6)	<0.001
8–10	46	(15.0)	31	(8.1)		53	(17.3)	27	(7.1)	
11 or more	36	(11.7)	8	(2.1)		50	(16.3)	15	(3.9)	
Missing	13	(4.2)	15	(3.9)		9	(2.9)	13	(3.4)	
Women										
0–7	100	(60.6)	156	(83.4)	<0.001	88	(53.3)	156	(83.4)	<0.001
8–10	31	(18.8)	20	(10.7)		35	(21.2)	22	(11.8)	
11 or more	30	(18.2)	4	(2.1)		38	(23.0)	4	(2.1)	
Missing	4	(2.4)	7	(3.7)		4	(2.4)	5	(2.7)	

COVID-19, coronavirus disease 2019; HADS, Hospital Anxiety and Depression Scale; IQR, interquartile range; PCC, post-COVID-19 condition.

The multivariable logistic analysis after adjustment for age, sex, comorbidities, severity of COVID-19 during hospitalization, BMI at admission, smoking status, years of education, household income, wave, and follow-up duration. The OR of HADS-Anxiety scores 8 or higher was 3.97 (95% CI, 2.76–5.70), and that of HADS-Depressive scores 8 or higher was 4.58 (95% CI, 3.24–6.46) (not shown in table).

## DISCUSSION

In this large-scale multicenter study of hospitalized COVID-19 patients, 45% of surviving patients had PCC 1 year after infection during the Alpha- and Delta-variant dominant waves, with a small variation in the prevalence of PCC between the two waves. The present study is the first to compare PCC between the two wave periods among hospitalized patients.

The common symptoms of PCC in our study included dyspnea, fatigue/malaise, muscle weakness, a decrease in concentration, and sleep disorder, followed by brain fog. In a meta-analysis of studies in Europe and China that included hospitalized and non-hospitalized patients, the prevalence of PCC approximately 1 year after infection between January 2020 and June 2020, the common symptoms were fatigue/weakness, myalgia, depression, anxiety, dyspnea/breathlessness, memory loss/memory complaints/forgetfulness, concentration/difficulties, insomnia/sleep difficulties, and joint pain.^4^ These symptoms were similar to our findings, albeit their proportions varied between our study and the meta-analysis.

In our study, we found that 19.8% of patients had HADS-Anxiety scores of 8 or more, and 23.5% of patients had HADS-Depression scores of 8 or higher. The portions of anxiety and depression, including suggestive symptoms, were four times higher in patients with PCC than without PCC. A previous survey of 1,969 patients hospitalized and discharged alive from hospitals in Spain during the first COVID-19 pandemic in March to May 2020 (the Wild-type dominant wave) showed that 15.7% of patients had 8 or higher scores of HADS-Anxiety and 19.0% had HADS-Depression scores of 8 or higher at 8.4 months after discharge.^18^ Research conducted in the Netherlands with 246 patients admitted to the ICU for COVID-19 between March and July, 2020 (the Wild-type dominant wave) revealed that 17.9% of patients had 8 or higher scores of HADS-Anxiety and 18.3% had 8 or higher scores of HADS-Depression 1 year after ICU treatment.^3^ These proportions were similar between our study and the previous studies despite the different timing of the Wild-type and the Alpha- and Delta-variant dominant waves.

Our multicenter study’s strengths include a large sample of hospitalized COVID-19 patients, a high follow-up rate, and comparisons of PCC and mental health between the Alpha- and Delta-variant dominant waves. This study had several limitations. First, the following biases could have affected the results: 1) recall bias because the survey was conducted 1 year after infection; 2) survival bias because only those who were discharged from the hospital and participated in the study; and 3) selection bias due to the exclusion of those who were deemed difficult to answer the questionnaire. Second, because there was no control group, we could not clarify whether the symptoms were due to COVID-19 infection or changes in the environment and lifestyle during the COVID-19 pandemic. Finally, as half of the respondents had severe cases, post-intensive care syndrome could influence the frequency of post-COVID-19 symptoms.^19^^,^^20^

In conclusion, the present large-scale, multicenter study revealed the status of PCC and mental status among hospitalized survivors between April and September 2021 under the Alpha- and Delta-variant dominant waves. Further follow-up is required to clarify the long-term impact of COVID-19 on PCC, mental health, complications, daily life, and socioeconomic status.

## SUPPLEMENTARY MATERIAL

eMaterial 1. Twenty medical institutions and their departments that participated in this study

eTable 1. Response rate of by collaborative research institutions
