# Supplementary material for: Post-COVID-19 Condition in Hospitalized Survivors After 1 Year of Infection During the Alpha- and Delta-variant Dominant Waves in Japan: COVID-19 Recovery Study II
Source: J Epidemiol. 2025 Jul 5;35(7):330–40. doi: 10.2188/jea.JE20240179 (PMC12162180; doi:10.2188/jea.JE20240179)
Supplement: Supplementary file 1 [file je-35-330-s001.pdf]

eMaterial 1. Twenty medical institutions and their departments that participated in this study

Department of Emergency and Critical Care Medicine , Kansai Medical University Medical Center; Disease Control and Prevention Center and AIDS Clinical Center, National Center for Global Health and Medicine; Division of Trauma and Surgical Critical Care, Osaka General Medical Center; Center for Respiratory Disease, Department of Respiratory Medicine, Japan Community Healthcare Organization Hokkaido Hospital; Department of Emergency and Critical Care Medicine, St. Marianna University Yokohama Seibu Hospital; Department of Critical Care and Emergency Medicine, Japanese Red Cross Maebashi Hospital; Infection Prevention and Control Department, Yokohama City University Hospital; Department of Emergency and Critical Care Medicine, Osaka Medical and Pharmaceutical University; Department of Emergency and Critical Care Medicine, St. Marianna University School of Medicine; Department of Cardiology, Naha City Hospital; Department of Emergency and Critical Care Medicine, Hitachi General Hospital; Department of Emergency and Critical Care, Tokyo Metropolitan Tama Medical Center; Department of Emergency and Critical Care Medicine, Chiba University Graduate School of Medicine; Tajima Emergency and Critical Care Medical Center, Toyooka Public Hospital; Department of Emergency and Critical Care Medicine, Hiroshima University Hospital; Department of Anesthesiology and Intensive Care Medicine, University of the Ryukyus Hospital; Department of Intensive Care Medicine, Tokyo Medical and Dental University; Department of Emergency and Critical Care Medicine, Shinshu University School of Medicine; Department of Respiratory Medicine, National Hospital Organization, Hokkaido Medical Center; Advanced Critical Care and Emergency Center, Yokohama City University Medical Center.

eTable 1. Response rate of by collaborative research institutions

Institutions	Prefectures	Recruited	Responded	Response rate
		n	n	(%)
Kansai Medical University Medical Center	Osaka	434	176	40.6
National Center for Global Health and Medicine	Tokyo	390	147	37.7
Osaka General Medical Center	Osaka	289	121	41.9
Japan Community Healthcare Organization Hokkaido Hospital	Hokkaido	265	118	44.5
St. Marianna University Yokohama Seibu Hospital	Kanagawa	160	79	49.4
Japanese Red Cross Maebashi Hospital	Gunma	234	78	33.3
Yokohama City University Hospital	Kanagawa	87	59	67.8
Osaka Medical and Pharmaceutical University	Osaka	101	53	52.5
St. Marianna University School of Medicine	Kanagawa	117	51	43.6
Naha City Hospital	Okinawa	160	38	23.8
Hitachi General Hospital	Ibaraki	50	34	68.0
Tokyo Metropolitan Tama Medical Center	Tokyo	49	33	67.3
Chiba University Graduate School of Medicine	Chiba	42	33	78.6
Toyooka Public Hospital	Hyogo	34	27	79.4
Hiroshima University Hospital	Hiroshima	24	20	83.3
University of the Ryukyus Hospital	Okinawa	16	11	68.8
Tokyo Medical and Dental University	Tokyo	18	10	55.6
Shinshu University School of Medicine	Nagano	12	9	75.0
Hokkaido Medical Center	Hokkaido	18	7	38.9
Yokohama City University Medical Center	Kanagawa	12	5	41.7
Total		2,512	1,109	44.1
